# Heat Treatment and Dynamic Mechanical Analysis Strain Sweep Effects on the Phase Structure and Morphology of an Fe-28Mn-6Si-5Cr Shape Memory Alloy

**DOI:** 10.3390/nano13071250

**Published:** 2023-04-01

**Authors:** Mihai Popa, Florin Popa, Bogdan Pricop, Nicanor Cimpoeșu, Nicoleta-Monica Lohan, Gabriel Kicsi, Bogdan Istrate, Leandru-Gheorghe Bujoreanu

**Affiliations:** 1 Faculty of Materials Science and Engineering, “Gheorghe Asachi” Technical University of Iași, Blvd. Dimitrie Mangeron 71A, 700050 Iași, Romaniabogdan.pricop@academic.tuiasi.ro (B.P.);; 2 Faculty of Materials and Environmental Engineering, Technical University from Cluj-Napoca, Blvd. Muncii, No. 103-105, 400641 Cluj-Napoca, Romania; 3 Faculty of Mechanical Engineering, “Gheorghe Asachi” Technical University of Iasi-Romania, Blvd. Dimitrie Mangeron, No. 61-63, 700050 Iași, Romania

**Keywords:** dynamic mechanical analysis, internal friction, storage modulus, differential scanning calorimetry, precipitation, shape memory alloy, martensite plates

## Abstract

Fe-Mn-Si-based shape memory alloys (SMAs) have been extensively investigated since 1982 for various useful properties that enhance the development of different applications such as anti-seismic dampers for very tall buildings, pipe joints, or rail fasteners. In particular, the Fe-28Mn-6Si-5Cr (mass. %) alloy has been mainly used in vibration mitigation or self-adjustable axial displacement applications. Dynamic mechanical analysis (DMA), performed by strain sweeps (SS), enables the monitoring of the evolution of storage modulus and internal friction variations with increasing strain amplitudes at different constant frequencies and temperatures. Thus, applying dynamic bending with various frequencies and amplitudes that actually represents an isothermal mechanical treatment. In the present paper, an Fe-28Mn-6 Si-5Cr (mass. %) SMA was cast by ingot metallurgy, hot-rolled, and water quenched in order to obtain thermally induced martensite and avoid the occurrence of cooling cracks. The influence of the holding time, between 2 and 10 h, at 1050 °C and the effects of DMA-SS performed at three different frequencies were analyzed by a differential scanning calorimetry, an X-ray diffraction, and a scanning electron and atomic force microscopy. The effects of the holding time and mechanical treatment on the structure and morphology of martensite plates were corroborated with the results of the thermal analysis.

## 1. Introduction

Fe-Mn-Si shape memory alloys (SMAs) have been extensively investigated in the last four decades due to their lower cost compared to NiTi-based SMAs [1], their superior mechanical properties, such as high recovery stresses, good machinability, weldability, or formability [2], and their fair corrosion resistance [3]. It has been pointed out that small Si additions to Fe-30Mn alloys (in mass. %, as all chemical compositions will be given hereinafter) promote a reversible martensitic transformation [4]. Thus, ε-hexagonal close-packed (hcp) stress-induced martensite retransforms to γ-face-centered cubic (fcc) austenite during heating, and this is the mechanism of the shape memory effect (SME) in Fe-Mn-Si SMAs [5]. The occurrence of α’ (body-centered cubic, bcc) martensite, observed at low Mn amounts or high deformation degrees, mostly in the intersection areas of ε-hcp martensite plates, delays the reverse martensitic transformation [6]. Aiming to increase the corrosion resistance, Cr was added, and, thus, Fe-28Mn-6Si-5Cr SMA was developed [7], which became of practical use for the construction of steel pipe couplings for fluid transport [8] or pipe joining in tunnel making [9], crane rail fasteners [10], and concrete reinforcing bars [11]. The feasibility of truncated cone modules being used for the self-adjustable axial loading of angular contact bearings, such as to restore the contact between balls and cage, was demonstrated [12]. In particular, Fe-28Mn-6Si-5Cr SMA has been mainly used in vibration mitigation applications [13], as it belongs to inter-phase boundary-type high-damping materials, in which internal friction (IF) is caused by the hysteretic movement of the interfaces during the stress-induced martensitic transformations [14]. Since both SME and IF are based on the reverse movement of the γ/ε interface [5], the relationship between these two phenomena and the stress-induced formation of ε-hcp martensite from γ-fcc austenite has been intensely studied in Fe-Mn-based SMAs [15]. Considering the necessity to accurately evaluate the elastic–plastic behavior and energy dissipation capacity of a given material, dynamic mechanical analysis (DMA) has been developed as a measuring technique for transformation temperatures of solid state transitions, storage modulus (E’), loss modulus (E”), and IF (tanδ = E”/E’) [16]. This technique comprises the dynamic loading of a specimen, performed during heating–cooling (temperature scans, TS) or isothermal strain sweeps (SS), with controlled force, deformation, frequency, and temperature. Various specimen holders may be used, among which three-point-bending (3PB) is one of the most popular [17].

Some previous studies on manganese (Hadfield) steel have reported that the DMA strain sweeps that are performed with a three-point-bending specimen holder (DMA-SS-3PB) are able to influence the microstructure that causes the formation of parallel arrays of micro-slip bands with submicronic spacing [18] and the preferred reordering tendency of carbon atoms [19]. On the other hand, DMA has been widely used as a measuring technique for the viscoelastic and damping properties of Fe-Mn-Si-based SMAs [20], while for Fe-28Mn-6Si-5Cr SMA, a strong influence of thermomechanical treatments on the IF values was reported [21]. Based on these results, the present authors focused their attention on Fe-28Mn-6Si-5Cr SMA and observed that, besides thermomechanical treatments, the IF values are also influenced by the dynamic deformation frequency [22]. Moreover, it has been demonstrated that DMA-SS-3PB, itself can be considered a mechanical treatment, since the storage modulus increases and saturates due to work hardening, while the IF experiences a local maximum [23].

The present paper aims to analyze the cumulative effects of the heat treatments, quantified by maintaining periods during the solution treatment, and the dynamic bending frequency, during DMA-SS-3PB, on the structure of an Fe-28Mn-6Si-5Cr SMA.

## 2. Materials and Methods

An Fe-28Mn-6Si-5Cr alloy was melted from high-purity elemental powders (over 99%) provided by Sigma Aldrich Merck (Darmstadt, Germany) in a FIVE CELLS levitation induction furnace (Lautenbach, France) and cast into water-chilled copper molds. From machined ingots, with typical dimensions of ϕ 20 × 45 mm, fragments were longitudinally cut and hot-rolled (1050 °C) until their thickness decreased to 1 mm. Further thickness reduction was achieved by intensive polishing under water cooling until reaching a 0.7 mm thickness. Rectangular specimens (0.7 × 4 × 25 mm) were cut on a wire spark erosion machine. The gradual change in the shape of manufactured specimens is illustrated in Figure S1 from Supplementary Material. Twenty-five samples were solution-treated at 1050 °C, a temperature that was used for the homogenization of this alloy [24]. During the solution treatment, every five samples were maintained for five different time durations: 2, 4, 6, 8, and 10 h, respectively, with final water quenching.

By means of a Setaram Labsys calorimeter (Setaram, Caluire, France), differential scanning calorimetry (DSC) tests were performed using small fragments, weighing less than 50 mg, and cut with a low-speed saw from the five solution-treated states. DSC charts were recorded both during heating, with a rate of 10 °C/min, up to 1050 °C, and during subsequent isothermal holding for 10 h.

Three samples of each of the above-mentioned five solution-treated states underwent DMA-SS-3PB by means of an analyzer-type DMA 242 Artemis NETZSCH (Netzsch, Selb, Germany), with force resolution of 0.0005 N, amplitude range: ±0.1 up to 240 μm, and amplitude resolution of 0.0005. Each of the three specimens was subjected to five consecutive cycles of strain sweep at room temperature (RT), with different frequencies: 1, 5, and 10 Hz, respectively, during which strain amplitude increased between 0.01 and 0.08%. Hereinafter, the specimens will be designated by their respective holding times and DMA-SS frequency (e.g., 2 h–1 Hz, 10 h–10 Hz). Considering that the first cycle usually corresponds to a transient phenomenon, it was removed from the diagrams, and the average values of E’ and tanδ were calculated from the second to the fifth cycle by means of PROTEUS software (v.6.1, Selb, Germany) that the DMA device is equipped with.

The structural effects of these treatments were evaluated by X-ray diffraction (XRD), scanning electron and atomic force microscopy (SEM and AFM).

XRD used an Expert PRO MPD diffractometer (Malvern Panalytical B.V., Almelo, Netherlands) with Cu Kα radiation. XRD patterns were recorded in the significance region 2θ = 40–100°. XRD maxima identification of γ-fcc, ε-hcp and α′-bcc phases was conducted using crystallographic databases and 01-071-8288, 01-071-8285, and 00-034-0396 JCPDS files, respectively. SEM micrographs were recorded on polished and etched surfaces with a JEOL JSM-5600LV device, which was equipped with an Oxford Instruments EDX (energy dispersive X-ray spectrometry) detector (INCA 200 software).

AFM micrographs were recorded with NanoSurf (Liestal, Switzerland) easyScan 2 equipment on electropolished surfaces. The morphologic particularities of martensite plates were investigated and measured, as previously pointed out [25].

## 3. Results

### 3.1. DSC Evaluation

**Figure 1:**
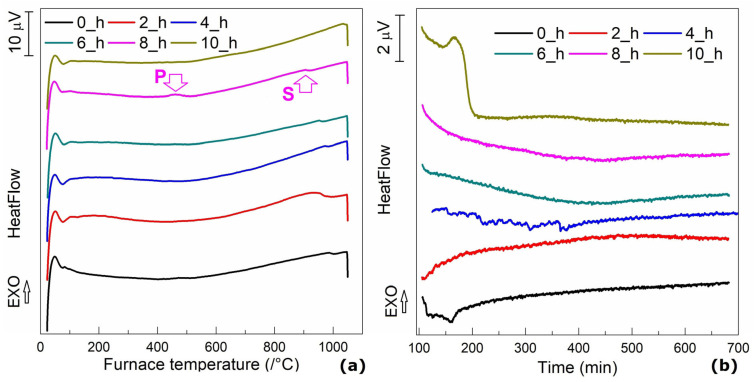
DSC thermograms emphasizing heat flow variations during the first two steps of solution treatment: (**a**) heating up to 1050 °C and (**b**) 10 h of holding at 1050 °C. P: precipitation; S: solvus.

Figure 1 separately summarizes the heat flow variations during the first two steps of the solution treatment: heating and holding.

On the DSC thermograms recorded during the heating of fragments cut from solution-treated specimens, Figure 1a revealed an exothermic peak located around 500 °C at the specimens solution treated for 8 and 10 h [23]. In the present case, due to the representation at the same temperature scale, the exothermic maximum is more obvious in the specimen solutions treated for 8 h. These peaks can be ascribed to a precipitation phenomenon. In addition, most of the DSC charts revealed an endothermic step, located around 900–950 °C, which corresponds to the solvus line [26]. It is noticeable from Figure 1b that the solution treatment’s holding time altered the heat flow variation during holding. The heat flow had an increasing tendency in time at specimens in the initial condition and solution-treated for 2 h, remained almost constant at the specimen held for 4 h, and experienced a decreasing tendency at specimens maintained for 6, 8, and 10 h.

### 3.2. DMA Evaluation

**Figure 2:**
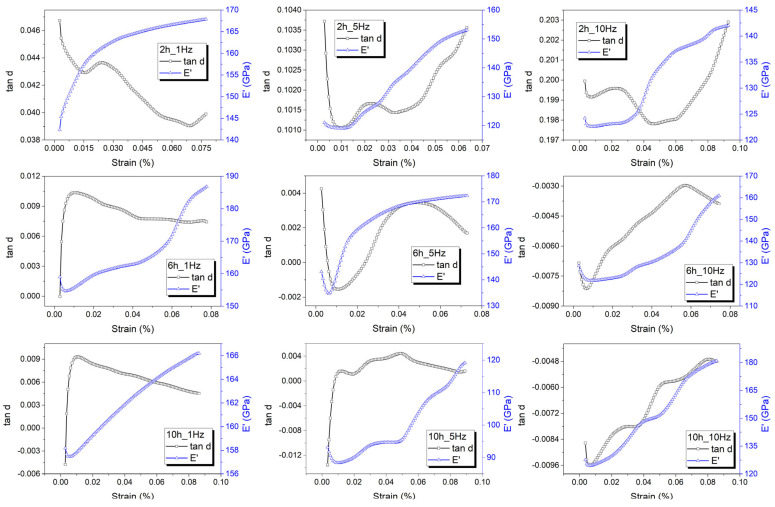
Variations of the average values of E’ and tanδ with strain amplitude as a function of holding time (2, 6, and 10 h, from **left** to **right**) and strain sweep frequency (1, 5, and 10 Hz, from **top** to **bottom**).

Figure 2 illustrates the variations in average values of E’ and tanδ with a strain amplitude for nine of the fifteen specimens under study.

In all of the diagrams, the storage modulus (E’) increased up to saturation, which was an effect of work hardening [23]. All the tanδ variations present a maximum that tends to increase with the DMA-SS-3PB frequency only for the specimens that were solution-treated for short holding times (2 h), and the same aspect was observed for the specimens solution-treated for 4 h. For longer holding times, it is expected that the precipitates might produce a “pinning effect” that blocks interface displacement [27].

### 3.3. SEM Investigations

**Figure 3:**
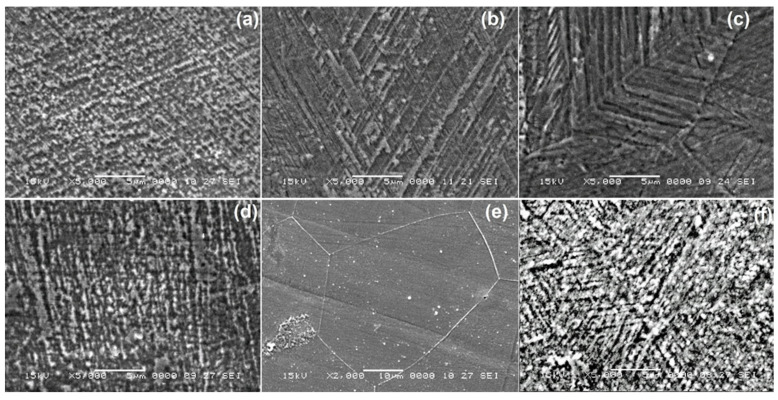
Typical SEM micrographs before DMA-SS-3PB: (**a**) in its initial hot-rolled state and at the specimens solution treated for: (**b**) 2 h; (**c**) 4 h; (**d**) 6 h; (**e**) 8 h; and (**f**) 10 h.

The characteristic SEM micrographs of the specimens in initial hot-rolled condition and after solution treatment, prior to DMA-SS-3PB, are summarized in Figure 3.

It is obvious that martensite plates can be observed neither in the initial hot-rolled state nor on the specimens that were solution-treated for 6, 8, and 10 h. Moreover, due to the precipitation emphasized in Figure 1a, the formation of martensite plates was completely hindered in Figure 3e. It can be concluded that thermally induced martensite formed only in the specimen solutions treated for 2 and 4 h.

**Figure 4:**
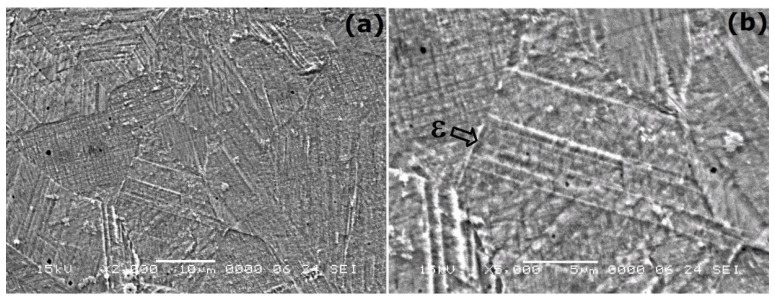
Typical SEM micrographs of a specimen in its initial hot-rolled state after DMA-SS-3PB: (**a**) general aspect; (**b**) detail of ε-hcp stress-induced martensite plates.

After the application of DMA-SS-3PB, the occurrence of stress-induced martensite can be noticed even in the specimens in their initial hot-rolled state that were not subjected to solution treatment, as illustrated in Figure 4.

The plates shown in Figure 4b have submicronic widths and straight shapes, which are characteristic to ε-hcp martensite. By comparing the microstructure in Figure 3a, which corresponds to the specimen in initial hot-rolled state, with the microstructure shown in Figure 4, it is obvious that DMA-SS-3PB caused the occurrence of ε-hcp martensite plates.

**Figure 5:**
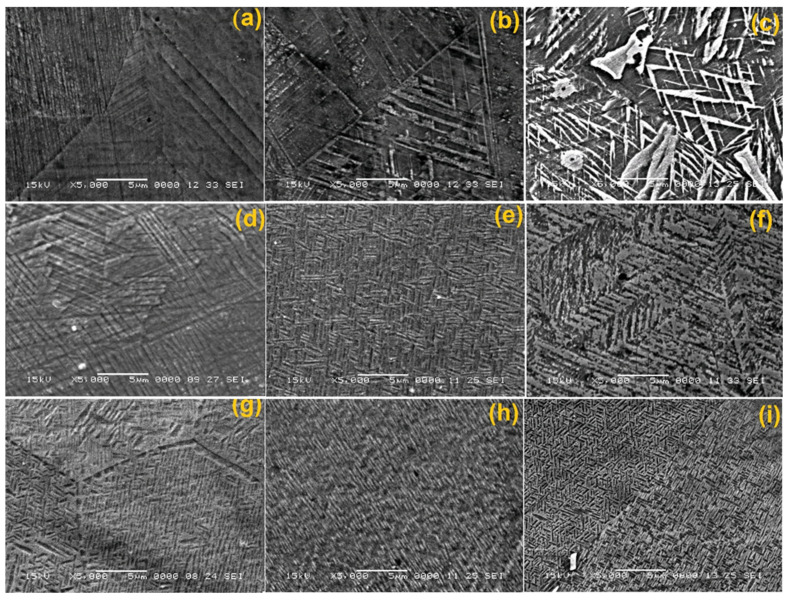
Typical SEM micrographs of solution-treated specimens after the application of DMA-SS-3PB: (**a**) 2 h–1 Hz; (**b**) 6 h–1 Hz; (**c**) 10 h–1 Hz; (**d**) 2 h–5 Hz; (**e**) 6 h–5 Hz; (**f**) 10 h–5 Hz; (**g**) 2 h–10 Hz; (**h**) 6 h–10 Hz; and (**i**) 10 h–10 Hz.

The effects of DMA-SS-3PB on the structure of solution-treated specimens are emphasized in the typical SEM micrographs shown in Figure 5.

Two phenomena are noticeable from analyzing the micrographs in Figure 5:

(i) The grain size tends to increase with an increasing holding time due to coalescence enhancement as an effect of minimizing grain boundary energy at the solid–solid interface [28].
(ii) Stress-induced martensite plates become shorter with increasing the frequency of DMA-SS-3PB. Straight thin plates are visible only at the specimen solutions treated for 2 h (Figure 5a,d,g).

**Figure 6:**
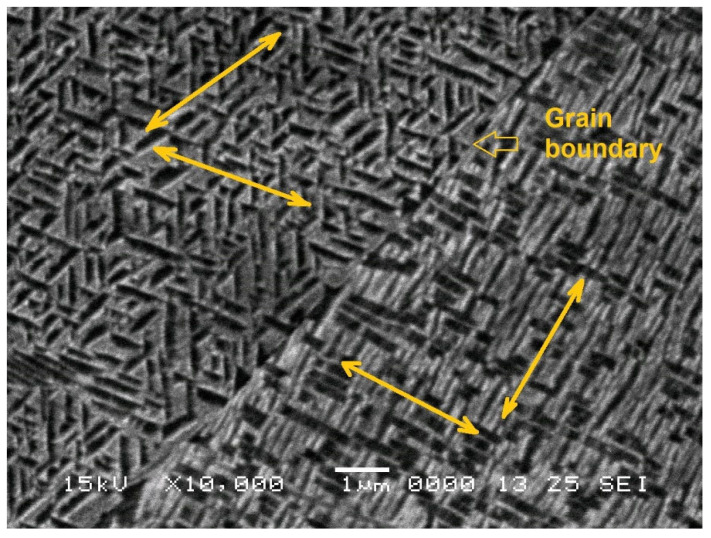
Detail of the SEM micrograph of specimen 10 h–10 Hz, from Figure 5i, emphasizing the crystallographic directions of the crystallites on both sides of a grain boundary.

With an increasing holding time, the crystallites tend to become wider and preferably oriented. Specimen 10 h–10 Hz, from Figure 5i shows very short and precisely oriented submicronic particles, as detailed in Figure 6.

It is obvious that the left-side particles are aligned along crystallographic directions tilted at approx. 60°, while those from the right side of the grain boundary make angles that are about 90°.

Considering that there were 5 DMA-SS-3PB cycles that each lasted for 14.9 min, it follows that the specimen 10 h–10 Hz was subjected to 14.9 min × 60 s × 10 oscillations/cycle × 5 cycles = 44,700 cycles. During this large number of cycles, martensite plates were stress-induced and fragmented to an average dimension of 0.2 × 1 μm. One can argue that for damping purposes, much larger numbers of cycles would be necessary within high-cycle fatigue testing. However, this is not the purpose of the present study.

### 3.4. XRD Investigations

**Figure 7:**
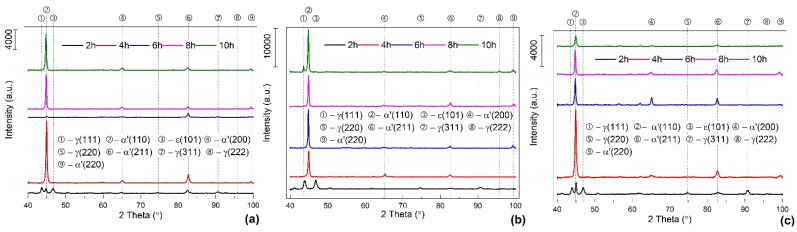
Typical XRD patterns of solution-treated specimens after the application of DMA-SS-3PB with different frequencies: (**a**) 1 Hz; (**b**) 5 Hz; and (**c**) 10 Hz.

XRD patterns were brought to the same scale and summarized in Figure 7.

By equalizing the XRD patterns, a more accurate qualitative analysis could be conducted with the single goal of emphasizing the occurrence of stress-induced martensite after DMA strain sweeps.

The crystallographic orientations (111), (220), and (311) are known for enhancing the stress-induced martensite reversion to austenite upon mechanical unloading [29], and for this reason, they are absent on the XRD patterns of the large majority of the specimens. Due to the accumulation of a large amount of dynamical deformation during the five DMA-SS-3PB cycles applied to each specimen, the formation of α’-bcc martensite was favored. In order to evaluate its unit cell deformation, the experimental values of its parameter, *a_exp_*, were calculated. Bragg’s Law was used to determine lattice spacings *d*_110_ (based on Cu wavelength λ_Cu_ = 0.1540598 nm and the experimental values of 2*θ*/2 angles). Then, *a_exp_* values were calculated based on lattice spacings and experimental Miller indices [30].

**Figure 8:**
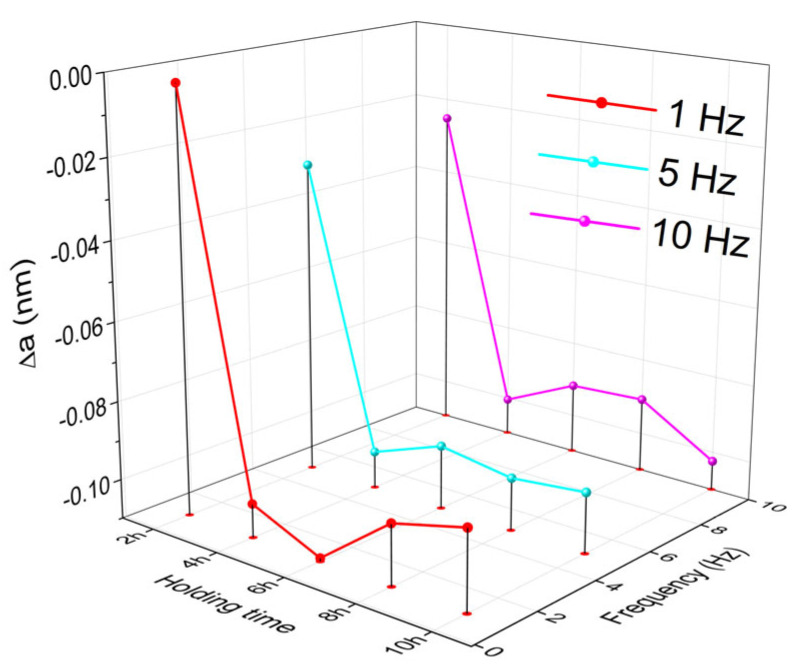
Variation of the unit cell deformation of α’-bcc martensite as a function of holding time and DMA-SS-3PB frequency, according to the XRD patterns from Figure 7.

Finally, the experimental value, *a_exp_*, was subtracted from the theoretical one, *a_theor_* = 0.3592 nm, given by the JCPDS files of 00-034-0396 crystallographic database. The variations of Δ*a* = *a_exp_* − *a_theor_*, as a function of the holding time and DMA-SS-3PB frequency, are shown in Figure 8.

It is obvious that, for each of the three DMA-SS-3PB frequencies, the unit cell of α’-bcc martensite was compressed by as much as a third of its initial value. It follows that the martensite is no longer cubic but becomes tetragonal after dynamical bending.

### 3.5. AFM Observations

**Figure 9:**
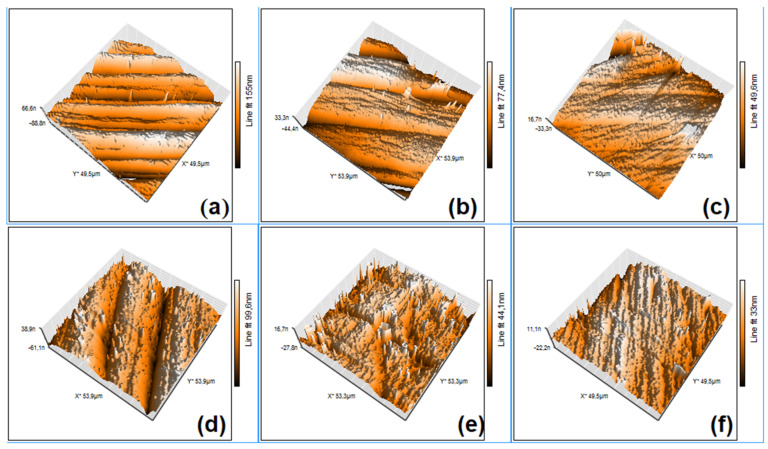
Typical AFM micrographs of solution-treated specimens, after the application of DMA-SS-3PB: (**a**) 2 h–1 Hz; (**b**) 2 h–5 Hz; (**c**) 2 h–10 Hz; (**d**) 10 h–1 Hz; (**e**) 10 h–5 Hz; and (**f**) 10 h–10 Hz.

For a better look at the changes induced by the holding time and strain sweep frequency, AFM observations were performed. Figure 9 shows the representative AFM micrographs of the specimens that were subjected to the shortest and longest holding times.

The AFM micrographs reveal martensite plate refinement with the increased holding time and the DMA-SS-3PB frequency. For the quantitative evaluation of the effects of the extreme values of the holding time and the deformation frequency on martensite plate dimensions, systematic measurements were performed. For this purpose, five characteristic groups (*i* = 1 ÷ 5) of martensite plates and five parallel plates (*j* = 1 ÷ 5) within each group were selected. Along each plate, the width and height of five equidistant profiles were measured, and the average values of the width (*w_ij_*) and height (*h_ij_*) were calculated for each plate along with the corresponding standard deviations. The results are listed in Table 1.

By averaging the values listed in Table 1, the mean heights and widths of the martensite plates can be determined for the four specimens that were processed under extreme holding time and deformation frequency conditions, as summarized in Table 2.

These values are in good agreement with the resultsFi obtained from SEM micrographs, and they demonstrate that the increase in both the holding time and deformation frequency caused the refinement of martensite plates. Nevertheless, the increase in the holding time from 2 to 10 h caused a decrease in the martensite plate width between 4.15 and 3.49 times and a decrease in their height between 3.97 and 3.71 times. Conversely, a frequency increase from 1 to 10 Hz caused plate width decreases of between 3 and 2.5 times and plate height decreases of between 2.9 and 2.7 times. These results prove that a holding time increase from 2 to 10 h is more effective than a deformation frequency increase from 1 to 10 Hz, from the point of view of martensite plate refinement.

## 4. Discussion

Finally, before ending the discussion of the above results, one should also consider the unique character of the martensite plates, which are stress-induced in Fe-Mn-Si-based SMAs during dynamic loading. It has been demonstrated that these SMAs experience the transformation of the martensite plate variants that reversibly change from tension-induced to compression-induced [31]. It can be assumed that this phenomenon also occurs during DMA strain sweep, when the specimens are alternatively bent by the analyzer’s pushrod.

It has been argued that stress-induced martensite plates should not intersect each other, and for this reason, it is recommended that they have small dimensions and a reduced number of crystallographic orientations [5]. Or this is exactly what the combination of 10 h of holding and a 10 Hz strain sweep has achieved, as pointed out by Figure 6 and Table 2.

The storage modulus reinforcement of the Fe-28Mn-6Si-5Cr SMA under this study, as an effect of dynamic strain sweep, is more desirable from the point of view of shape recovery stresses than from the point of view of vibration mitigation.

## 5. Conclusions

By summarizing the results of the investigations performed by DSC, DMA, SEM, XRD, and AFM, the following conclusions can be drawn concerning the effects of the solution treatment holding time and RT dynamical deformation frequency:

1. A precipitation phenomenon was identified by DSC in the specimens that were solution-treated for 8 and 10 h;
2. Dynamical deformations by RT strain sweeps caused work hardening and a general increase in the storage modulus;
3. At the precipitate-free specimens, which were solution-treated for 2 and 4 h, internal friction ranged between 0.1 and 0.2;
4. Neither in the initial hot-rolled specimens nor in the specimens that were solution-treated between 6 and 10 h, the martensite plates were not visible on SEM micrographs;
5. The application of RT strain sweeps caused the occurrence of stress-induced martensite or the fragmentation of the thermally induced one;
6. In solution-treated specimens, the increase in the holding time (from 2 to 10 h) was more effective than the increase in the deformation frequency (from 1 to 10 Hz) in the refinement of martensite plates;
7. After 10 h of holding at 1050 °C and the strain sweep application at a frequency of 10 Hz, the martensite plates reached the minimum mean values of their width (659 nm) and height (382 nm) and became preferably oriented;
8. The recorded XRD patterns indicated that after the dynamic deformation, most of the microstructure comprises mostly α’-bcc martensite, which was due to the high amount of dynamical deformation induced by the RT strain sweep;
9. A holding time increase from 2 to 10 h enhanced the compression of the parameter of the cubic unit cell by approx. 30%, which changed the crystallization system from cubic to tetragonal-centered cubic.
10. A new mechanical treatment has been introduced for Fe-Mn-Si-based SMAs, based on RT isothermal dynamic bending, which is able to reinforce the storage modulus and refine the martensitic structure.

## Figures and Tables

**Table 1:** Experimental values of martensite plate widths and heights (nm).

Specimen	Plate Group No.	Plate No.	Dimension	Average Value	Standard Deviation	Specimen	Plate Group No.	Plate No.	Dimension	Average Value	Standard Deviation
2 h–1 Hz	1	1	w_11_	16,244	770.2	2 h–10 Hz	1	1	w_11_	791	89.3
			h_11_	8639	675.8				h_11_	571	147
		2	w_12_	19,184	582.9			2	w_12_	626	66.1
			h_12_	11,404	463.4				h_12_	458	106.3
		3	w_13_	7376	800.7			3	w_13_	3037	353.6
			h_13_	4174	585				h_13_	1803	369.6
		4	w_14_	6797	2997.5			4	w_14_	446	46.4
			h_14_	4856	699.6				h_14_	278	19.4
		5	w_15_	5538	470.7			5	w_15_	597	120.8
			h_15_	3921	344.2				h_15_	264	74.6
	2	1	w_21_	4795	431.4		2	1	w_21_	686	140.2
			h_21_	2798	219.3				h_21_	404	133.7
		2	w_22_	4776	128.3			2	w_22_	687	111.4
			h_22_	2972	216.2				h_22_	437	107.4
		3	w_23_	4961	350.8			3	w_23_	837	123.9
			h_23_	3478	455.2				h_23_	462	50.1
		4	w_24_	2225	258.6			4	w_24_	8173	1257.5
			h_24_	1267	275.6				h_24_	6040	898.9
		5	w_25_	11,878	794.5			5	w_25_	13,300	645.9
			h_25_	7030	641.9				h_25_	7539	852.5
	3	1	w_31_	14,440	460.4		3	1	w_31_	10,412	820.7
			h_31_	8279	721				h_31_	6912	603.3
		2	w_32_	6519	435.5			2	w_32_	11,463	2182.3
			h_32_	4303	571.7				h_32_	6475	658.2
		3	w_33_	2302	345.1			3	w_33_	669	247.7
			h_33_	1696	102.4				h_33_	432	168.6
		4	w_34_	6403	510.7			4	w_34_	661	122.9
			h_34_	3960	89				h_34_	288	72.4
		5	w_35_	7784	306.6			5	w_35_	544	69.9
			h_35_	3852	262.2				h_35_	369	55.6
	4	1	w_41_	9855	534.8		4	1	w_41_	738	243.7
			h_41_	5458	648.7				h_41_	475	184.1
		2	w_42_	4131	272			2	w_42_	606	187.7
			h_42_	3237	287.3				h_42_	366	127.4
		3	w_43_	3764	420.4			3	w_43_	563	194.1
			h_43_	4041	949.4				h_43_	335	193.1
		4	w_44_	6286	421.4			4	w_44_	397	54.4
			h_44_	3700	372.3				h_44_	237	47.6
		5	w_45_	10,320	264			5	w_45_	328	86.4
			h_45_	5889	254				h_45_	264	77.3
	5	1	w_51_	7146	674.9		5	1	w_51_	399	84.6
			h_51_	394	422.2				h_51_	218	22.9
		2	w_52_	4085	240.7			2	w_52_	340	68.3
			h_52_	3456	188.6				h_52_	221	27.6
		3	w_53_	4066	531.2			3	w_53_	432	60.5
			h_53_	2574	1312.5				h_53_	254	64
		4	w_54_	2361	206.6			4	w_54_	448	57.7
			h_54_	1891	160.5				h_54_	230	39.4
		5	w_55_	3631	318.7			5	w_55_	660	40.5
			h_55_	2245	251.3				h_55_	311	47.3
10 h–1 Hz	1	1	w_11_	6355	1265.5	10 h–10 Hz	1	1	w_11_	848	128.6
			h_11_	4225	166.5				h_11_	505	106.4
		2	w_12_	3896	440.7			2	w_12_	939	73
			h_12_	2574	181.8				h_12_	454	49
		3	w_13_	2999	516.8			3	w_13_	713	96.6
			h_13_	1608	169.6				h_13_	937	61
		4	w_14_	2460	320.9			4	w_14_	744	66
			h_14_	1732	225.8				h_14_	476	24.2
		5	w_15_	2529	480.5			5	w_15_	859	42.7
			h_15_	1432	311.4				h_15_	480	28.7
	2	1	w_21_	3665	361.4		2	1	w_21_	673	13.2
			h_21_	2801	70.8				h_21_	381	53.2
		2	w_22_	3056	210.1			2	w_22_	658	82.5
			h_22_	2273	187.9				h_22_	391	32
		3	w_23_	1059	55.8			3	w_23_	737	82
			h_23_	526	54				h_23_	402	40.1
		4	w_24_	983	57.5			4	w_24_	999	127.2
			h_24_	501	66.8				h_24_	516	85.2
		5	w_25_	763	81.9			5	w_25_	842	100.3
			h_25_	433	49.7				h_25_	485	65.6
	3	1	w_31_	620	185.9		3	1	w_31_	695	40.5
			h_31_	380	118.5				h_31_	429	28.8
		2	w_32_	694	74.4			2	w_32_	694	66
			h_32_	416	25.5				h_32_	430	30.4
		3	w_33_	619	88.9			3	w_33_	808	80.5
			h_33_	361	47				h_33_	527	101.9
		4	w_34_	740	72			4	w_34_	782	22.6
			h_34_	381	39.5				h_34_	417	28.5
		5	w_35_	866	80.6			5	w_35_	664	140.1
			h_35_	471	43.8				h_35_	327	73.3
	4	1	w_41_	791	49.5		4	1	w_41_	327	24.8
			h_41_	428	50				h_41_	188	24.7
		2	w_42_	936	48.7			2	w_42_	350	37.5
			h_42_	466	41.5				h_42_	194	22.4
		3	w_43_	2032	110.2			3	w_43_	824	210
			h_43_	1044	57				h_43_	387	65.9
		4	w_44_	798	166.3			4	w_44_	706	31.1
			h_44_	533	146.8				h_44_	336	41.5
		5	w_45_	1779	150.6			5	w_45_	707	57.1
			h_45_	911	90.1				h_45_	345	42.5
	5	1	w_51_	1235	51.4		5	1	w_51_	326	67.2
			h_51_	775	63.7				h_51_	144	20
		2	w_52_	1118	169.6			2	w_52_	311	52.1
			h_52_	687	61.2				h_52_	188	42.3
		3	w_53_	948	94.3			3	w_53_	714	34.9
			h_53_	524	37.4				h_53_	339	20.7
		4	w_54_	884	50.1			4	w_54_	870	54.6
			h_54_	518	59.3				h_54_	395	57.7
		5	w_55_	665	65.8			5	w_55_	340	74.4
			h_55_	406	23.9				h_55_	177	40

**Table 2:** Mean values of martensite widths (w_mean_) and heights (h_mean_) determined from Table 1 (nm).

Specimen	2 h–1 Hz	2 h–10 Hz	10 h–1 Hz	10 h–10 Hz
w_mean_	6913	2298	1664	659
h_mean_	4119	1417	1037	382

## Data Availability

Not applicable.

## Supplementary Materials

The following supporting information can be downloaded at: https://www.mdpi.com/article/10.3390/nano13071250/s1, Figure S1: The gradual change in the shape of manufactured specimens.
