# Supplementary figures and images for: Heat Treatment and Dynamic Mechanical Analysis Strain Sweep Effects on the Phase Structure and Morphology of an Fe-28Mn-6Si-5Cr Shape Memory Alloy
Source: Nanomaterials (Basel). 2023 Apr 1;13(7):1250. doi: 10.3390/nano13071250 (PMC10096908; doi:10.3390/nano13071250)

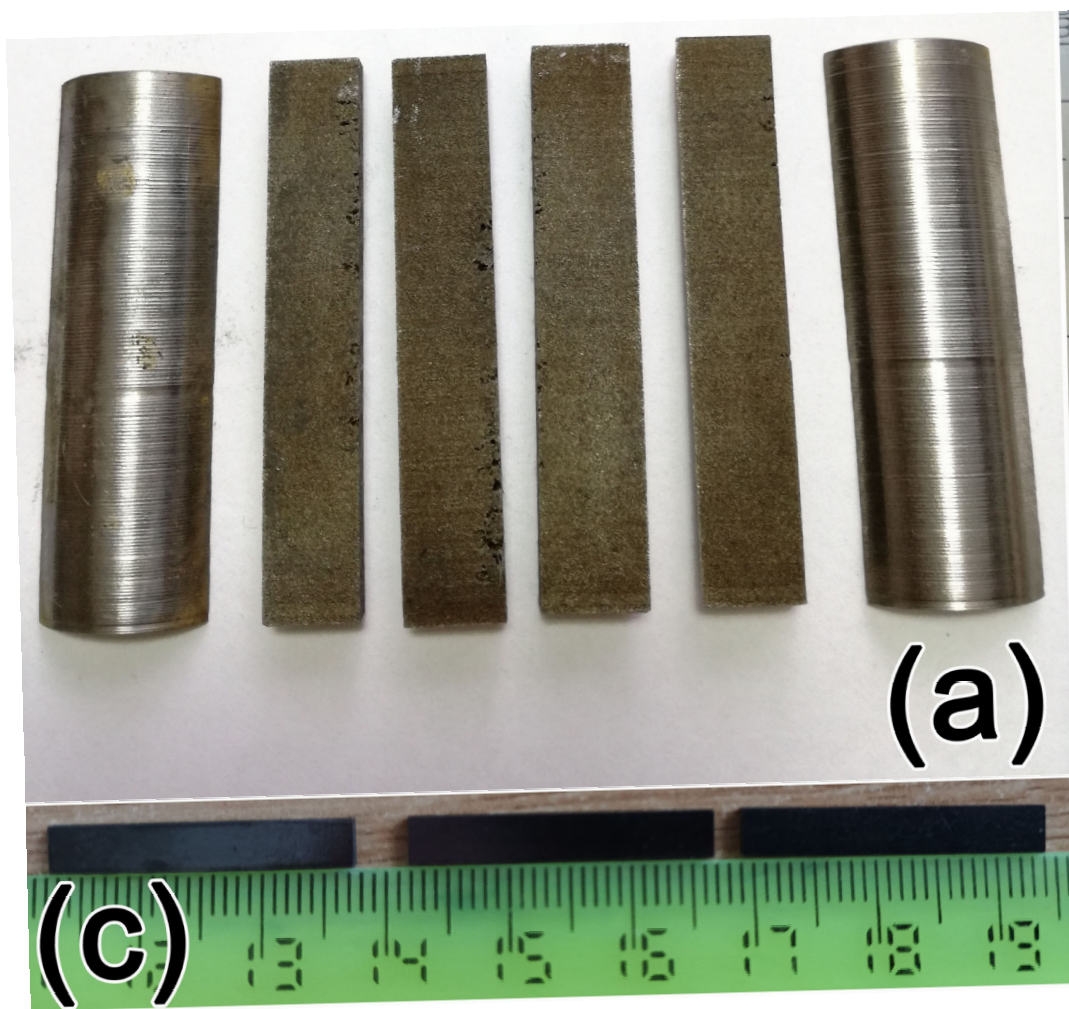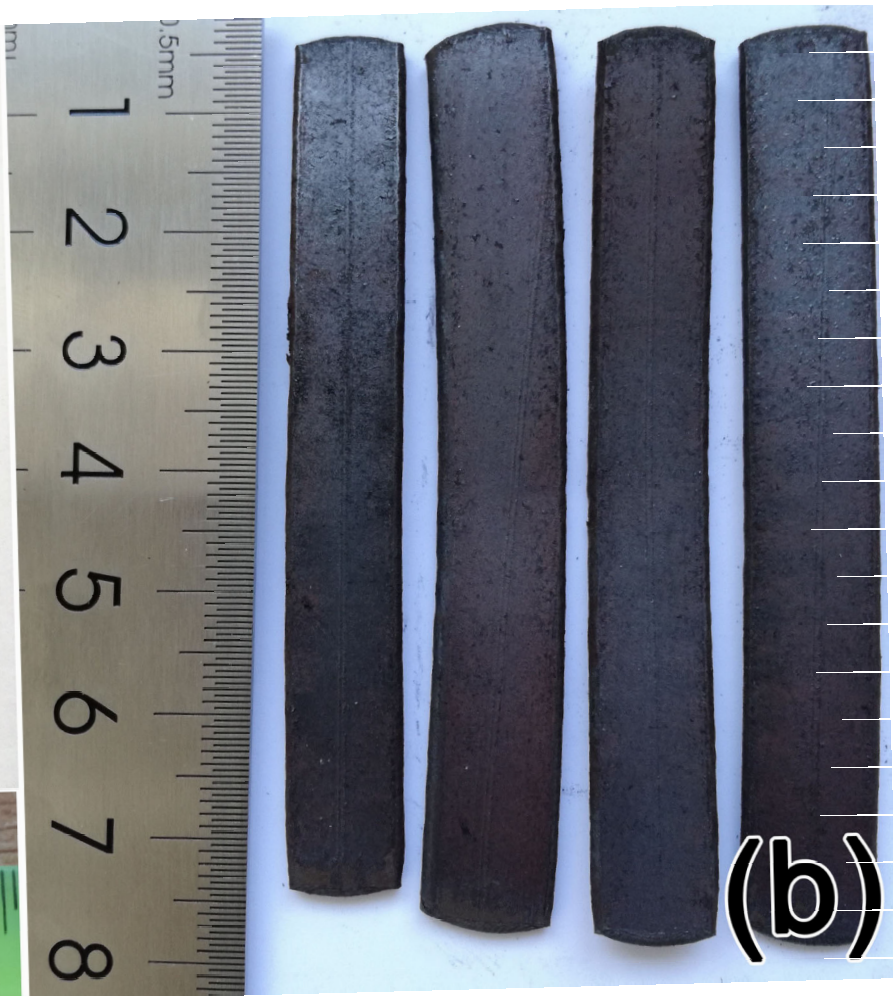

Supplement: Supplementary file 1 [file nanomaterials-13-01250-s001.zip › Figure S1.pdf]
